# Synthetic Mimics of Wound-Induced Carrot MicroRNAs Affect Lipid Metabolism in Cultured Adipocytes

**DOI:** 10.3390/nu17182919

**Published:** 2025-09-10

**Authors:** Edwin E. Reza-Zaldívar, Daniel A. Jacobo-Velázquez

**Affiliations:** 1 Tecnologico de Monterrey, Escuela de Ingenieria y Ciencias, Ave. General Ramon Corona 2514, Zapopan 45138, Jalisco, Mexico; edwin.reza@tec.mx; 2 Tecnologico de Monterrey, Escuela de Medicina y Ciencias de la Salud, Ave. Ignacio Morones Prieto 3000, Monterrey 64710, Nuevo León, Mexico

**Keywords:** cross-kingdom gene regulation, plant-derived microRNAs, adipogenesis, lipolysis, nutritional epigenetics, carrot (*Daucus carota*), obesity

## Abstract

**Background/Objectives:** Plant-derived microRNAs (miRNAs) have emerged as cross-kingdom regulatory molecules, but their capacity to influence mammalian metabolism is still poorly understood. This study aimed to investigate whether miRNAs induced in carrots (*Daucus carota*) by postharvest wounding stress can modulate adipocyte lipid accumulation. **Methods:** High-throughput small RNA sequencing was performed to identify stress-responsive miRNAs in wounded carrots. Bioinformatic analyses predicted potential mammalian targets, focusing on genes involved in adipogenesis and lipid regulation, including those in the insulin and FoxO signaling pathways. Selected miRNAs were functionally validated in 3T3-L1 adipocytes by assessing intracellular triglyceride levels and glycerol release. **Results:** Six stress-responsive carrot miRNAs were predicted to target mammalian lipid metabolism genes. Functional assays revealed that miR165a-3p, miR232a-5p, and miR1799 significantly decreased intracellular triglyceride accumulation and increased glycerol release, suggesting enhanced lipolysis. These effects support the potential regulation of adipocyte metabolism through plant miRNA mimics. **Conclusions:** Our findings provide experimental evidence for stress-induced carrot miRNAs mimics potentially modulate fat accumulation. This work expands current understanding of dietary plant miRNAs and highlights their potential role as functional food components for metabolic health improvement. Further research is needed to establish their gastrointestinal stability, uptake from dietary matrices, and in vivo effects.

## 1. Introduction

Plant-based diets are widely recognized for their health-promoting potential, including a reduced risk of obesity and metabolic disorders. These benefits have traditionally been attributed to the high content of vitamins, fiber, and phytochemicals in fruits and vegetables. Carrots (*Daucus carota*), for example, are rich in β-carotene (provitamin A), dietary fiber, and polyphenols, compounds linked to improved metabolic health and protection against chronic diseases [1]. Beyond conventional nutrients, recent insights suggest that plants may also contribute to human health through less orthodox bioactive components, such as dietary microRNAs (miRNAs) derived from plant foods.

MicroRNAs are ~20–24 nucleotide non-coding RNAs that regulate gene expression post-transcriptionally. The miRNAs typically bind to the 3′ untranslated regions of target mRNAs to repress translation or induce mRNA degradation, thereby influencing processes from development to metabolism. Plants likewise utilize miRNAs to regulate their own gene networks, especially under stress conditions [2]. Notably, plant miRNAs have a distinctive feature, a 2′-O-methylated 3′ end that improves resistance to degradation, enhancing their stability during food processing and gastrointestinal digestion. This structural modification supports the possibility that dietary plant miRNAs can persist after ingestion and exert biological effects [3,4,5]. Despite differences in biogenesis, plant and animal miRNAs share functional parallels, raising the intriguing possibility that plant miRNAs might exert biological effects across kingdom boundaries when ingested by animals.

Initially, miRNAs were believed to only function within their species of origin. However, increasing evidence has triggered a debate about mammalian gene regulation through plant miRNAs acquired from their diet [6]. This phenomenon, referred to as cross-kingdom gene regulation, is supported by preclinical and clinical studies demonstrating the absorption and functionality of plant miRNAs in mammalian systems [7,8]. For example, Zhang et al. [9] reported that a rice-derived miRNA (MIR168a) was detectable in the blood of mice fed a rice-based diet and could bind to and downregulate a mammalian gene (LDLRAP1) involved in cholesterol metabolism. This finding provided initial evidence that plant miRNAs from food might survive digestion and influence gene expression in consumers. Subsequent research has added support to this cross-kingdom regulation hypothesis. For instance, Chin et al. [10] demonstrated that oral administration of a plant miRNA (miR159) in mice inhibited breast cancer growth, coinciding with the miRNA’s uptake and direct silencing of the oncogenic transcription factor TCF7 in the tumor. More recently, Díez-Sainz et al. [11] showed that a peach-derived miRNA (miR6262) can modulate human gene expression related to lipid metabolism and thermogenesis in liver and fat cells, highlighting the potential for dietary plant miRNAs to impact metabolic pathways in the consumer. While not all studies agree on the bioavailability of plant miRNAs, these examples underscore the potential of cross-kingdom gene regulation as a novel mechanism by which plant-based diets could influence human health.

Carrots represent an appealing model to investigate this phenomenon because they are a common dietary vegetable with known health benefits and a well-characterized repertoire of endogenous miRNAs. Like other plants, carrot expresses numerous miRNAs, including ones that respond to environmental stresses [12]. Importantly, the biological content of carrots can be altered by stress; postharvest wounding (such as shredding) is known to trigger a stress response in the carrot tissue, leading to increased production of secondary metabolites and potentially changes in miRNA expression. Indeed, plant miRNAs have been identified as part of wound and defense responses in various species [2]. Additionally, computational analyses suggest that carrot-derived miRNAs may target key human genes involved in metabolic regulation—for example, recent in silico work found carrot miRNAs could interact with genes in the BMP/SMAD, C/EBP, and KLF signaling pathways that underlie obesity and metabolic syndrome [13]. This raises the question of whether carrot miRNAs, especially those induced under stress conditions, might exert functional effects on mammalian cells related to obesity or metabolism.

Based on this background, we hypothesize that carrot-derived miRNAs, particularly those upregulated by wounding stress, can act as cross-kingdom regulators of gene expression in adipocytes and thereby influence lipid metabolism in a way that combats obesity. To test this hypothesis, the present study integrated bioinformatic and experimental approaches. We first performed high-throughput small RNA sequencing to identify miRNAs that are induced in carrots by wounding stress. Next, using target prediction algorithms and pathway enrichment analysis, we identified putative human gene targets of these carrot miRNAs and examined which biological pathways they might regulate. Finally, we selected several wound-induced carrot miRNAs and conducted in vitro functional assays by transfecting them into 3T3-L1 adipocytes, a well-established mouse cell model of fat cells, to evaluate their effects on lipid accumulation. By combining these approaches, our study aims to determine whether carrot-derived miRNAs can modulate adipocyte fat metabolism, providing new insights into how plant-based diets may exert cross-kingdom molecular interactions.

## 2. Materials and Methods

### 2.1. Wounding Stress Application in Carrots

Fifteen kilograms of commercial-grade carrots without visible defects were selected for wounding stress experiments as previously described [14]. Carrots were rinsed with tap water, disinfected with a chlorine solution (200 ppm, pH 6.5–7.0) for 5 min, then dried with absorbent paper towels. The carrots were peeled by hand and shredded using a mechanical food processor (Waring Commercial, Model WFP11, Torrington, CT, USA) to apply wounding stress. Manual spinning in a salad spinner removed excess moisture from shredded carrots. Samples were stored at 20 ± 2 °C, and subsamples were collected at 24 and 48 h for RNA extraction. All samples were subsequently preserved at −80 °C until total RNA isolation.

### 2.2. Total RNA Isolation

RNA extraction was performed using a TRIzol-based (Cat. T9424 Merck, Burlington, MA, USA) method following the manufacturer’s instructions. Carrot samples were frozen in liquid nitrogen, and approximately 300 mg of each tissue was ground into a fine powder and placed into 1.5 mL tubes. Following that, 1 mL of TRIzol reagent was added, vortexed, incubated for 5 min at RT, and then centrifuged at 12,000× *g* for 10 min at 4 °C. The supernatant was transferred to a new tube, and 200 µL of cold chloroform (Cat. 650498 Merck, Burlington, MA, USA) was added, vortexed, and incubated at RT for 10 min. After centrifugation, the supernatant was mixed with 500 µL of chilled isopropanol (Cat. 190764 Merck, Burlington, MA, USA) and vortexed. After a 10 min incubation on ice, the mixture was centrifuged again (12,000× *g*, 10 min, 4 °C). The pellet was washed twice with 1 mL of pre-cooled 75% ethanol (Cat. 459844 Merck, Burlington, MA, USA), air-dried, and resuspended in RNase-free water (Cat. 10977035 Invitrogen, Waltham, MA, USA). RNA purity was assessed using the OD 260/280 (≥1.8) ratio with a Nanodrop spectrophotometer.

### 2.3. Small RNA Seq

Sequencing libraries were constructed using 1 μg of RNA per sample and SMARTer smRNA-Seq Kit according to the manufacturer’s protocol. Library sequencing was carried out with the Illumina HiSeq platform and single-end sequencing. Once the sequencing data, the Illumina universal adaptors were trimmed from the raw reads with the command-line interface cutadapt v1.1.2.1 according to the SMARTer smRNA-Seq Kit manufacturer’s instructions, discarding reads with lengths of less than 18 nucleotides. Reads with an average base quality/Phred score < Q20 were removed using fqtrim v0.9.7. Sequence alignment against miRBase v22 was performed using Bowtie2 v.1.1.1 and the miRDeep2 software package v 0.1.3, allowing a maximum of one mismatch and up to 200 instances of multi-mapping (m = 200). Finally, a text file containing the miRNAs read counts was generated.

The DESeq2 v 2.11 was used to analyze the DEG for samples applying thresholds of Fold Change ≥ 2.00 and padj ≤ 0.5.

### 2.4. Homo Sapiens Gene Target Prediction

Human target genes were predicted using the psRNAtarget (https://www.zhaolab.org/psRNATarget/, accessed on 17 July 2024) web server 162 (2017 release), a tool for determining the reverse complementarity between small RNA and target-site of target transcript and assessing the target accessibility by computing unpaired energy [15]. The mature carrot miRNA sequences were queried for human gene target prediction against the cDNA library of *Homo sapiens* transcripts available on the web server. The default setting was used in the V2 scoring schema; the maximum expectation was set to 5. Lower values provide more stringent prediction results, but more potential target sequences might be missed. Considering 5 as the expectation value is ideal to get acceptable targeting results, while an expectation over 5 may give unreliable targeting results. The penalty for the G: U pair was set to 0.5, the penalty for other mismatches was set to 1, and the penalty for the opening gap was set to 2. Moreover, the number of mismatches allowed in the seed region was set to 2 (mismatches of more than 2 in seed regions give rise to unreliable targeting results). Translation inhibition was set between 9 and 11 nucleotides, and the length for complementarity scoring size was set to 19. V2 schema assumes a priori that UPE does not influence the miRNA–target sequence interaction [16]. This strategy with permissive parameters is a sensitivity-first, hypothesis-generating approach in the emerging field of cross-kingdom miRNA research. It prioritizes sensitivity over specificity to avoid missing weak but potentially meaningful interactions.

### 2.5. Signaling Pathways Analysis

The list of predicted targets was submitted to a functional annotation tool provided by the Database for Annotation, Visualization, and Integrated Discovery release v2025_1 (DAVID https://davidbioinformatics.nih.gov/, accessed on 19 July 2024) [17] and annotated by the Kyoto Encyclopedia of Genes and Genomes (KEGG, Release 114.0, accessed on 19 July 2024) pathways, with redundant pathways manually removed. The pathway was significantly enriched if it passed the count threshold of three genes per annotation term and presented an EASE score with a Benjamini–Hochberg correction set to <0.05. In the DAVID database, the EASE score is a modified Fisher exact *p*-value used for enrichment analysis within gene lists, with *p*-value = 0 representing perfect enrichment. For subsequent in vitro studies, only miRNAs with predicted targets linked to adipose tissue metabolism, adipocyte browning, and/or lipid metabolism were selected.

### 2.6. Pulsed Stem-Loop RT-qPCR

From the identified miRNA, nine were further validated using stem-loop PCR and qPCR. Selected stem-loop RT primers were designed according to Chen et al. [18] and Kramer [19]. Sequence data are presented in Table 1. The pulse stem-loop RT was performed as described by Varkonyi-Gasic & Hellens [20] with slight modifications. Briefly, 25 µM of the appropriate stem-loop RT primer (Table 1) was initially denatured by heating to 65 °C for 5 min and then incubated on ice for 2 min. The RT master mix (Cat. 18090050, Invitrogen, Waltham, MA, USA) was prepared by mixing 1 μL of 10 mM dNTP mix (Cat. AM8228G Invitrogen, Waltham, MA, USA), 2 μL of denatured stem-loop RT primer (25 µM), and 1 µg of total RNA. The mix was centrifuged and heated to 65 °C for 5 min. Then, it was mixed with 4 μL 5X SSIV buffer, 1 μL 0.1M DTT, 1 μL RNaseOUT (40 U/μL), 1 μL superScript IV RT (200 U/μL) (Cat. 18090050, Invitrogen, Waltham, MA, USA), and nuclease-free water up to 20 µL. The cDNA synthesis settings were an initial incubation for 30 min at 16 °C, followed by pulsed RT of 60 cycles at 30 °C for 30 s, 42 °C for 30 s, and 50 °C for 1 s, and finally, an incubation at 85 °C for 5 min to inactivate the reverse transcriptase.

The SYBR green qPCR assay was done by mixing 4 μL SYBR Green master mix (Cat. 100029284, Invitrogen, Waltham, MA, USA),1 μL 10 μM forward miRNA-specific primer (Table 1), 1 μL 10 μM of the universal reverse primer, and 4 μL RT product. The qPCR settings were UDG activation at 50 °C for 2 min, reaction activation at 95 °C for 2 min, followed by 35 cycles of 95 °C for 15 s, 60 °C for 15 s, and 72 °C for 1 min. The relative expression levels of the miRNAs were calculated according to the 2^−ΔΔCt^ method using the U6 small nuclear RNA as a housekeeping control.

### 2.7. Cell Culture and Transfection of Cells with miRNAs

The 3T3-L1 cell line (ATCC, Manassas, VA, USA) was cultured under standard conditions with DMEM with high glucose (Cat. D7777 Merck, Burlington, MA, USA) supplemented with 10% calf serum (Cat. 35-054CM Corning, Corning, NY, USA). The culture was maintained in an incubator at 37 °C and a 5% CO_2_ atmosphere. The mature plant miRNA mimics novel-hsa-miR-376a-5p UUAGAUUCGCGCACAAACUCmG; novel-oan-miR-1421ag-5p CGGUGGACUGCUUGAGCUGmC; novel-hpo-miR-232a-5p UCCGCAGUAGCACUGGCmU; novel-rno-miR-337-3p UUCAGCUCACAUAUCUCUCUGmG; novel-ath-miR165a-3p UCGGACCAGGCUUCAUUCCCmC; novel-gga-miR-1799 GGAAGUGAUGAGUAGACmU; and a scramble control 5′-GGGACUAGCGGUUAGUGAUAmG-3, synthesized by Integrated DNA Technologies (m denotes 2-O-methylation at the 3′), were transfected into 3T3-L1 cells using the HiPerFect Transfection Reagent (Cat. 301704 Qiagen, Germantown, MD, USA) following manufacturer’s instructions. In brief, 3T3-L1 cells were seeded at a density of 1–2 × 10^5^ cells per well in DMEM supplemented with 10% calf serum and incubated overnight at 37 °C under 5% CO_2_. After reaching confluency, cells were transfected with 200 pMol/well of miRNA mimics and a scramble control.

### 2.8. T3-L1 Cell Differentiation

Transfected 3T3-L1 cells were differentiated to evaluate the potential anti-obesogenic effect of carrot miRNA mimics. Differentiation process was induced with DMEM-high glucose supplemented with 10% fetal bovine serum, 0.5 mM isobutyl-methyl-xanthine, 1 μM dexamethasone, and 5 μg/mL insulin (Cat. I5879, D4902, and I0516, Merck, Burlington, MA, USA) [19]. On the fourth day of the differentiation protocol, the cell medium was switched to DMEM-high glucose supplemented with 10% fetal bovine serum and 10 μg/mL insulin. On the seventh day, the differentiation medium was changed to DMEM-high glucose supplemented with 10% fetal bovine serum. Cells were considered fully differentiated on day 12.

### 2.9. Oil Red O Staining

The oil red O staining procedure allows visual and quantitative identification of the lipids present within the lipid vacuoles of differentiated adipocytes [21]. Following 12 days of 3T3-L1 differentiation induction, the culture medium was removed, and the cell monolayer was washed twice with 0.01 M PBS at pH 7.4. Subsequently, the cells were fixed with 4% PFA for 15 min, washed with 0.01 M PBS, and then with 60% isopropanol (IPA). An oil red O working solution (5 mg/mL in IPA) was added and incubated for 20 min at RT for staining. Thereafter, the monolayer was washed thrice with 0.01 M PBS and observed under a microscope (OPTIKA IM-3, OPTIKA, Ponteranica, BG, Italy) for photographic documentation (OPTIKA PRO8 Digital Camera C–P8, OPTIKA, Italy). Finally, the dye was extracted with 60% IPA. Total lipid content was determined spectrophotometrically at 490 nm. For analysis, the absorbance of each treatment was compared with that of the negative control.

### 2.10. Free Glycerol Measurement

Free glycerol levels were assessed following the differentiation of 3T3-L1 cells using a colorimetric kit (Cat. AB133130, Abcam, Cambridge, UK) following the manufacturer’s instructions. Briefly, 25 μL of cell culture supernatant and 100 μL of Free Glycerol Assay Reagent were added to a 96-well plate and incubated for 15 min. The absorbance was measured at 540 nm. Glycerol content was quantified using a standard curve. Results were expressed as a percentage relative to the control group.

### 2.11. Triglycerides Measurement

Triglyceride accumulation was assessed post-differentiation of 3T3-L1 cells with a lipase-based kit (Cat. AB65336, Abcam, Cambridge, UK). In brief, harvested cells were resuspended in 1 mL of a 5% Triton X-100 solution, heated to 90 °C in a water bath for 5 min, and subsequently cooled to room temperature. This procedure was repeated twice. The solution was centrifuged at 10,000× *g* for 2 min, and the supernatant was transferred to a new tube and diluted 10-fold. Subsequently, 2 μL of cholesterol esterase/lipase solution was added and incubated for 20 min at RT. Finally, 50 μL of Triglyceride Reaction mix was added and incubated for 60 min in the dark. Triglycerides were quantified by measuring absorbance at 570 nm. Results were expressed as a percentage relative to the control group.

### 2.12. Statistical Analysis

The difference between the groups in culture assays was determined by the one-way analysis of variance (ANOVA), applying the Tukey post hoc test to analyze multiple comparisons. Data analysis was performed using the GraphPad Prism v10.4.2 software, and a statistically significant difference was considered when the value of *p* < 0.05. Results are presented as the mean ± SE. Differential expression analysis of miRNAs was performed with DESeq2 v 2.11, considering a Fold Change ≥ 2.00 and *p*-value ≤ 0.05 as screening criteria.

## 3. Results

### 3.1. Wounding Stress Alters Carrot miRNA Profiles

**Figure 1:**
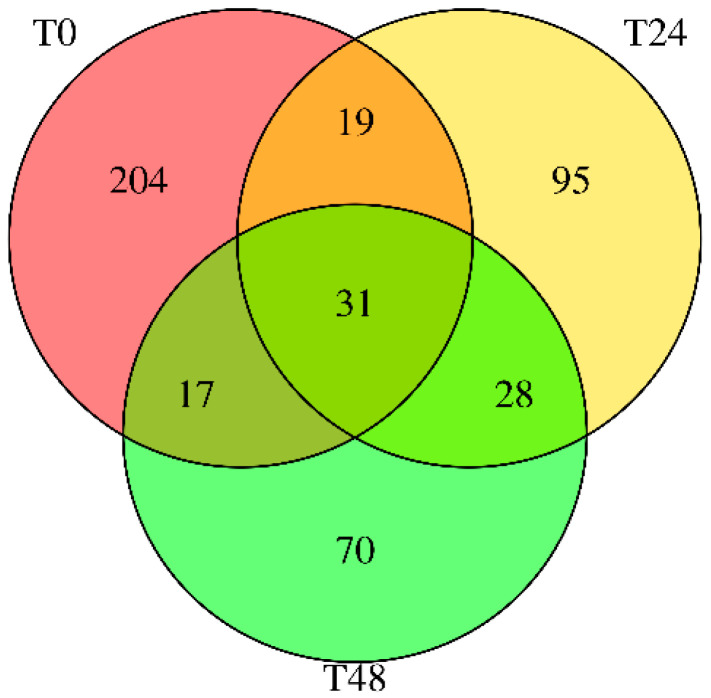
Venn diagram of carrot miRNAs identified in the three libraries. T0 = shredded tissue with no incubation. T24 = shredded tissue with 24 h of incubation at 20 ± 2 °C. T48 = shredded tissue with 48 h of incubation at 20 ± 2 °C.

The small RNA sequencing of wounded carrot tissues revealed a dynamic miRNA expression landscape (Figure 1). Across all samples, 464 distinct carrot miRNAs were detected, underscoring the complexity and plasticity of plant small RNA regulation [16]. However, the number of active miRNAs declined with prolonged stress: fresh-cut carrots (T0) expressed 204 miRNAs, while those incubated for 24 h (T24) and 48 h (T48) showed only 95 and 70 miRNAs, respectively (Sequence IDs in Supplementary File S1). Only 31 miRNAs persisted in all three conditions. This progressive loss of miRNA diversity suggests that wounding and incubation selectively repress certain miRNAs, potentially through degradation or shifts in transcriptional priorities under stress. Similar patterns of stress-induced miRNA modulation have been reported in other plants, such as salt-stressed carrots [12], *Arabidopsis thaliana* under drought and heat stress [2,22], and tomato under osmotic stress [23]. In each case, specific miRNAs are attenuated to reallocate resources for survival.

**Figure 2:**
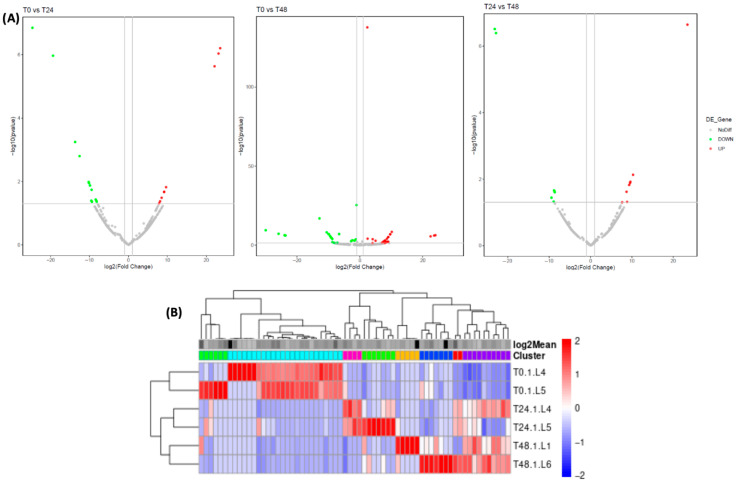
Differential Expression Analysis of carrot miRNAs (**A**) Volcano plot of differentially expressed miRNAs. Each point in the volcano plot represents a gene, the horizontal axis represents the logarithm of the difference in the expression of a gene in two samples, and the vertical axis represents the negative logarithm of the *p*-value. The larger the absolute value of the horizontal axis, the greater the difference in the expression level between the two samples. The larger the vertical axis value, the more significant the differential expression, and the more reliable the differentially expressed genes. (**B**) Cluster analysis of differentially expressed miRNAs. Red color represents genes with higher expression, while green color represents genes with lower expression. Different columns represent different samples, while different rows represent different miRNA.

Differential expression (DE) analysis confirmed that wounding elicits pronounced changes in miRNA levels over time (Figure 2). At 24 h post-wounding, 23 miRNAs were differentially expressed relative to T0 (14 up-regulated, 9 down-regulated), and by 48 h, 55 miRNAs showed significant changes (24 up, 31 down). A direct comparison of 24 h vs. 48 h indicated 15 miRNAs continuing to shift (8 higher at 24 h, 7 higher at 48 h). The increase in certain miRNAs after 48 h, but not at 24 h, can be explained by the temporal dynamics of stress perception and signaling in plants [24]. Upon exposure to stress, plants initiate early signaling pathways (e.g., ROS bursts, Ca^2+^ influx, hormone signaling) within minutes to hours. However, many stress-responsive miRNAs are secondary regulators, whose expression depends on transcriptional reprogramming and the accumulation of intermediate signals. In summary, these results (Figure 2, Supplementary File S2) illustrate a wave of miRNA reprogramming following the initial wounding: some miRNAs surge transiently at 24 h before subsiding, while others require prolonged stress to change. Such temporal modulation aligns with the idea that plants fine-tune miRNA expression as an early stress response and again during sustained stress. Indeed, this mirrors prior observations in *Daucus carota* under salinity stress, where distinct sets of miRNAs respond at early vs. late stress phases [12].

**Figure 3:**
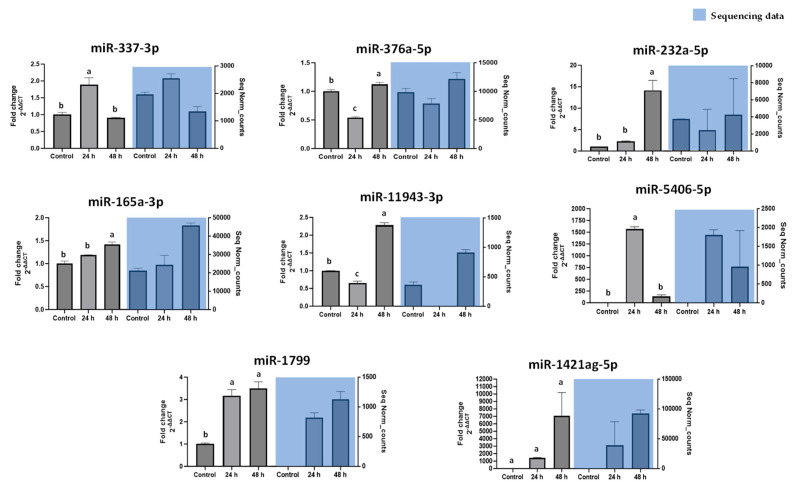
Relative expression level by qPCR of selected carrot miRNAs contrasting the small-RNA seq data. The data shown are the mean ± S.E. Different letter denote a statistically significant difference *p* ≤ 0.05.

Notably, key miRNAs identified as most responsive to wounding were validated by stem-loop RT-qPCR. Figure 3 shows that the qPCR data corroborated the sequencing trends. For example, novel-ath-miR165a-3p, one of the most up-regulated miRNAs after 24 h, exhibited a significant increase by qPCR, whereas novel-rno-miR-337-3p, which showed minimal change by sequencing, remained low by qPCR as well. This validation highlights a subset of wound-responsive miRNAs (including miR165a-3p, novel-hpo-miR-232a-5p, novel-gga-miR-1799, among others) that undergo robust expression changes under abiotic stress. Consistency between sequencing and qPCR (Figure 3) confirms the reliability of our high-throughput data and pinpoints candidate carrot miRNAs for further functional analysis.

### 3.2. Cross-Kingdom Target Predictions of Carrot miRNAs

**Figure 4:**
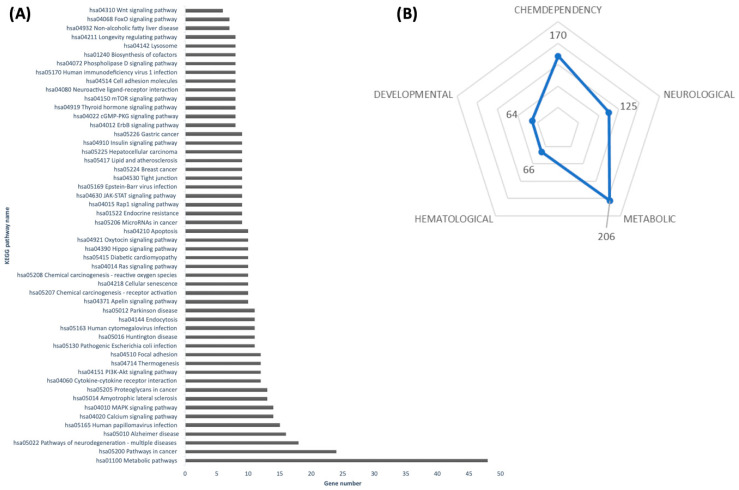
Functional annotation of the predicted gene target with DAVID. (**A**) Annotated gene targets in KEGG pathways database. (**B**) Annotated gene targets in GAD disease class. All terms were considered significant considering at least seven hits and *p*-value < 0.05.

Emerging evidence suggests that dietary plant miRNAs can survive digestion and regulate gene expression in animals [9,10]. In light of this cross-kingdom hypothesis, we bioinformatically examined whether carrot miRNAs might target genes in mammals. For this study, we prioritized six wound-induced carrot miRNAs (novel-hsa-miR-376a-5p, novel-oan-miR-1421ag-5p, novel-hpo-miR-232a-5p, novel-rno-miR-337-3p, novel-ath-miR165a-3p, and novel-gga-miR-1799) from the 31 persistent candidates. The selection was based on higher read counts across all stress conditions, with preliminary bioinformatic predictions indicating a potential regulation of a larger number of mammalian genes compared with other candidates, and enrichment of their predicted targets in signaling pathways directly implicated in obesity-related processes, such as insulin signaling, FoxO signaling, Wnt signaling, and lipid metabolism. The six carrot miRNAs aligned against the human transcriptome using the psRNATarget algorithm [15] (Supplementary File S3). Strikingly, each carrot miRNA was predicted to bind numerous human mRNAs, enriching in pathways central to metabolism and disease (Figure 4A). For example, targets fell within KEGG pathways for general metabolism, insulin and Wnt signaling, thermogenesis, FoxO signaling, and even longevity regulation.

Such pathways are major regulatory hubs of energy balance and cell fate. Indeed, FoxO transcription factors are pivotal integrators of insulin signaling, stress responses, and aging, orchestrating processes from glucose homeostasis to adipogenesis [25]. Our in silico results suggest that carrot-derived miRNAs (e.g., the wound-induced miR165a-3p and miR376a-5p) could potentially mimic or modulate these conserved pathways in humans by binding to the same network nodes.

To further explore the health implications, we annotated the predicted gene targets for disease associations. Using DAVID and the Genetic Association Database (GAD), many target genes were linked to metabolic disorders, including obesity, dyslipidemia, and cardiovascular disease (Figure 4B), as well as pathways implicated in inflammation and oxidative stress [17]. This suggests any cross-kingdom activity of carrot miRNAs might be especially relevant to metabolic health.

Taken together, these computational findings expand the nutritional significance of carrots beyond vitamins and fiber. They raise the possibility that carrot miRNAs act as bioactive dietary components capable of fine-tuning metabolism at the molecular level, echoing reports of other plant miRNAs influencing mammalian physiology [11,26]. As others have proposed, enriching diets or supplements with specific plant miRNAs could be a novel strategy to modulate disease pathways. Our results provide candidate carrot miRNAs and target genes to test in that context.

### 3.3. Carrot miRNAs Suppress Lipid Accumulation in Adipocytes

**Figure 5:**
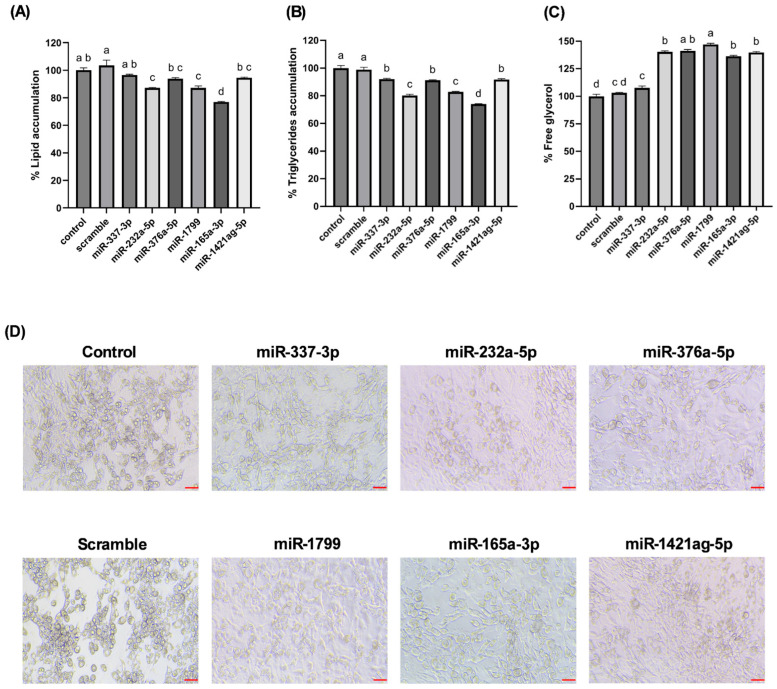
Effect of the carrot miRNAs after 3T3-L1 differentiation. (**A**) Quantification of Oil Red O staining, (**B**) triglycerides quantification, (**C**) free glycerol quantification, and (**D**) representative images of adipocytes transfected with a specific carrot miRNA. The data shown are the mean ± S.E. Each different letter denotes a statistically significant difference *p* ≤ 0.05. The scale bar is 100 μm.

From the wound-responsive miRNAs, six were prioritized for functional studies based on their persistence across stress conditions, higher read counts relative to T0, and predicted targets. To directly assess cross-kingdom functionality, we transfected several wound-induced carrot miRNAs into mouse 3T3-L1 adipocytes and measured their effects on fat storage. Remarkably, cells treated with carrot miRNA mimics (miR165a-3p, miR232a-5p, miR1799) accumulated significantly less lipid during differentiation compared to control cells. By day 12 post-transfection, Oil Red O staining revealed a 13–23% reduction in lipid droplet accumulation (Figure 5A) in miRNA-treated adipocytes. Consistently, triglyceride assays showed up to a 26% decrease in intracellular triglyceride content relative to controls (Figure 5B). In both readouts, novel-ath-miR165a-3p elicited the strongest anti-adipogenic effect, while novel-rno-miR-337-3p, a carrot miRNA not induced by wounding, had no significant effect.

Concomitantly, adipocytes exposed to carrot miRNAs exhibited increased lipolysis. Free glycerol released into the culture medium, a byproduct of triglyceride breakdown, was over 40% higher in cells treated with the effective miRNAs than in controls, indicating enhanced fat catabolism (Figure 5C). Novel-gga-miR-1799 was particularly potent, increasing free glycerol by approximately 47%, while miR-337-3p again showed no change. Microscopic images of the adipocytes (Figure 5D) visually confirmed these findings: cells transfected with miR165a-3p or miR1799 appeared markedly less lipid-laden (fewer and smaller Oil Red O-stained droplets) than control cells, consistent with a leaner phenotype.

These preliminary functional assays suggest that certain carrot miRNAs can interfere with adipocyte fat accumulation. Although the precise mechanisms remain under investigation, the observed phenotypes likely involve downregulation of pro-adipogenic genes or signaling pathways. For instance, miR165a-3p and others may target transcripts within the insulin or Wnt signaling cascades that drive adipogenesis [13]. By analogy, a recent study demonstrated that a maize miRNA (miR167e-5p) promotes adipogenesis by repressing β-catenin, thus lifting the Wnt-mediated inhibition of fat cell differentiation [27].

In contrast, the carrot miRNAs studied here produced the opposite outcome, reduced lipid stores, implying they may target genes that promote lipogenesis or differentiation. Supporting this idea, miR6262 from peach was shown to suppress RXRA and other nuclear receptors in fat cells, leading to reduced expression of lipid-storage genes such as *PLIN1*, and increased markers of thermogenesis [11]. This result is broadly consistent with our observations of reduced triglyceride content and smaller lipid droplets in 3T3-L1 adipocytes treated with carrot miRNAs.

Although the specific gene targets of carrot miRNAs in adipocytes remain to be experimentally validated, the phenotypic effects observed here strongly suggest functional cross-kingdom regulation of metabolic genes in mammalian adipose tissue. These results provide a promising foundation for future studies aimed at uncovering the molecular interactions between dietary miRNAs and host adipogenic pathways.

## 4. Discussion

Our findings highlight a potential connection between the stress-induced miRNA profile of carrots and lipid accumulation in cultured adipocytes. Wounding carrots by shredding not only triggers a general decline in total miRNA species but also selectively boosts certain miRNAs (e.g., miR165a-3p, miR232a-5p, miR1799) that appear to carry bioactivity across kingdoms. The abundance changes measured in Figure 3, directly translated into functional outcomes when the miRNA mimics were introduced into mammalian cells. The results provide in vitro evidence that certain carrot miRNA mimics suppressed lipid accumulation in 3T3-L1 adipocytes (Figure 5). This supports a potential causal chain: carrot wounding → increased specific miRNAs → miRNA uptake by adipocytes → reduced fat storage. In essence, the plant’s stress response may enhance its health-promoting potential by concentrating miRNAs with lipid-modulating properties.

Importantly, our cross-kingdom approach offers a novel and complementary perspective to previous research on the health benefits of wound-stressed carrots. Reza-Zaldívar et al. [14] reported that shredded carrots incubated for 24 h exhibited enhanced anti-obesity and anti-inflammatory activity in vitro and in animal models. Although that study was conducted using methanolic extracts, thus excluding microRNAs as active components, our findings suggest that miRNAs induced under similar post-harvest conditions could represent an additional, previously unexplored mechanism of action. By targeting mammalian genes involved in adipogenesis and lipid metabolism, carrot-derived miRNAs may function as regulators fat cell development. Our in silico pathway analysis indicated that these miRNAs could modulate critical metabolic regulators, such as insulin and FoxO signaling pathways, which govern adipocyte differentiation and lipid homeostasis [25]. When delivered to adipocytes, carrot miRNA mimics effectively downregulated lipid storage (Figure 5), mimicking the outcomes expected from inhibition of pro-adipogenic signals. This observation aligns with emerging evidence that plant-derived dietary miRNAs can influence mammalian gene expression and metabolic phenotypes [9,10]. It also complements findings by Díez-Sainz et al. [11], who showed that miR6262 from peach reprogrammed gene expression in human adipocytes—downregulating RXRA and PLIN1, key genes in lipid storage—even if the phenotypic changes (e.g., browning or lipid loss) were modest. In addition to modulating adipogenic signaling, plant miRNAs may also influence cholesterol flux pathways. A recent study reported that dietary plant miRNAs regulate cellular cholesterol efflux by targeting transporters such as ABCA1 and ABCG1 [26]. This suggests that the carrot-derived miRNAs identified here, some of which were predicted to interact with lipid transport and metabolism genes, may extend their influence beyond triglyceride regulation to cholesterol handling. Although our work did not directly evaluate these pathways, this possibility warrants further exploration in future transcriptomic and functional assays.

In contrast to our results, a study by Chen et al. [18] demonstrated that miR167e-5p from maize enhanced lipid accumulation in 3T3-L1 cells by repressing β-catenin, thereby de-repressing downstream adipogenic transcription factors like PPARγ. This juxtaposition underscores that not all dietary plant miRNAs have the same biological effects. Their functional outcome depends on the identity of their mammalian gene targets. While miR167e-5p lifts repression of adipogenesis, our carrot miRNA mimics may instead target components that promote lipogenesis or differentiation, thereby suppressing fat cell development. Both studies implicate the Wnt/β-catenin–PPARγ axis, but in opposite directions, revealing the complexity and specificity of cross-kingdom regulatory interactions. Further research is needed to establish their mechanistic and in vivo effects.

These functional differences open the door to precision nutrition strategies. By selectively consuming foods or extracts rich in anti-adipogenic plant miRNAs, one might be able to modulate fat formation in vivo. Although in vitro models like 3T3-L1 cannot fully capture the biological complexities of digestion, absorption, and tissue distribution, our data present a compelling proof of concept that plant miRNA mimics can modulate core metabolic pathways.

From a practical standpoint, our results suggest an accessible dietary intervention: shredding carrots and allowing them to incubate for 24 h enhances their miRNA content, especially for miRNAs with anti-lipogenic effects.

Interestingly, plant miRNAs are known to be chemically stable, owing to their 2′-O-methylated 3′ ends and packaging into vesicles, making them more resilient than many other dietary bioactives during cooking and gastrointestinal transit [9,28]. However, more research is needed to determine the optimal post-harvest conditions for miRNA enrichment, as well as to verify miRNA absorption, biodistribution, and in vivo efficacy.

Our study contributes to a growing paradigm shift in nutrition science: food is not just a source of macronutrients and vitamins, but also of regulatory molecules, including miRNAs. Although the concept of cross-kingdom gene regulation via diet remains debated, accumulating evidence supports the idea that specific plant miRNAs can influence mammalian physiology [10,11]. Our data add to this literature by linking stress-induced carrot miRNAs to tangible metabolic effects in a mammalian cell model.

The broader implications are exciting. One could imagine breeding or engineering vegetables to overexpress health-promoting miRNAs, or developing functional foods and supplements that deliver targeted miRNAs to regulate human metabolic pathways. Future diets might be designed not only for nutrient content but also for regulatory RNA composition. In this context, the humble carrot, viewed through the lens of miRNA biology, offers a striking example of how a common food can be leveraged for cross-kingdom metabolic benefits.

## 5. Conclusions

In conclusion, this study demonstrates that miRNAs from a common dietary plant can exert regulatory control over mammalian cell metabolism. Carrot-derived miRNAs upregulated by post-harvest wounding stress were shown to attenuate adipogenesis and promote lipid catabolism in 3T3-L1 adipocytes. Specifically, transfection of carrot miRNA mimics (such as miR165a-3p, miR232a-5p, and miR1799) led to reduced fat accumulation and increased glycerol release, signifying a shift toward enhanced lipolysis. These findings provide evidence of a cross-kingdom regulatory mechanism, bridging plant stress physiology with mammalian metabolic regulation. The novelty of our work lies in identifying plant miRNAs (focusing on functional bioactive dietary components) that can influence adipocyte biology. By establishing a link between carrot wounding-induced miRNA profiles and anti-adipogenic effects in mammalian cells, we propose a new nutritional paradigm in which plant-derived small RNAs contribute to the health benefits of plant-based diets. This insight has significant practical relevance; it suggests that beyond traditional nutrients and phytochemicals, plant miRNAs could be harnessed as natural, food-based tools to modulate pathways involved in lipid metabolism and adipose tissue development. Further research, particularly in vivo studies, will be essential to determine the bioavailability of these plant miRNAs and to explore their potential in dietary strategies for managing obesity and metabolic disorders.

## Figures and Tables

**Table 1:** Primer Sequences Used for Pulsed Stem-Loop RT-qPCR Validation of Carrot-Derived miRNAs.

miRNA ID		Primer Sequence
novel-hsa-miR-376a-5p	miRNA mature sequence	UUAGAUUCGCGCACAAACUCG
	stem-loop primer	GTCGTATCCAGTGCAGGGTCCGAGGTATTCGCACTGGATACGACCGAGTT
	Forward	GTG CCC TTA GAT TCG CGC
novel-ptc-miR396f	miRNA mature sequence	UUCCACGGCUUUCUUGAACUG
	stem-loop primer	GTCGTATCCAGTGCAGGGTCCGAGGTATTCGCACTGGATACGACCAGTTC
	Forward	GCC CCT TCC ACG GCT TTC
novel-aca-miR-5406-5p	miRNA mature sequence	UUUUUAGACCAAAGGCUCGCU
	stem-loop primer	GTCGTATCCAGTGCAGGGTCCGAGGTATTCGCACTGGATACGACAGCGAG
	Forward	GCC GCC GTT TTT AGA CCA AT
novel-oan-miR-1421ag-5p	miRNA mature sequence	CGGUGGACUGCUUGAGCUGC
	stem-loop primer	GTCGTATCCAGTGCAGGGTCCGAGGTATTCGCACTGGATACGACGCAGCT
	Forward	CTC CGG TGG ACT GCT TG
novel-hpo-miR-232a-5p	miRNA mature sequence	UCCGCAGUAGCACUGGCU
	stem-loop primer	GTCGTATCCAGTGCAGGGTCCGAGGTATTCGCACTGGATACGACAGCCAG
	Forward	CCT GCT CCG CAG TAG CA
novel-rno-miR-337-3p	miRNA mature sequence	UUCAGCUCACAUAUCUCUCUGG
	stem-loop primer	GTCGTATCCAGTGCAGGGTCCGAGGTATTCGCACTGGATACGACCCAGAG
	Forward	GGC CCC TTC AGC TCA CAT
novel-ath-miR165a-3p	miRNA mature sequence	UCGGACCAGGCUUCAUUCCCC
	stem-loop primer	GTCGTATCCAGTGCAGGGTCCGAGGTATTCGCACTGGATACGACGGGGAA
	Forward	GAT CTC TCG GAC CAG GCT
novel-gga-miR-1799	miRNA mature sequence	GGAAGUGAUGAGUAGACU
	stem-loop primer	GTCGTATCCAGTGCAGGGTCCGAGGTATTCGCACTGGATACGACAGTCTA
	Forward	GGT CGC GGA AGT GAT GAG
novel-pte-miR-11943-3p	miRNA mature sequence	UUAUGGUGAUUUAUUUGUGUGG
	stem-loop primer	GTCGTATCCAGTGCAGGGTCCGAGGTATTCGCACTGGATACGACCCACAC
	Forward	GGG GGC GTT ATG GTG ATT T
U6 reference	Forward	ACAGAGAAGATTAGCATGGCC
	Reverse	GACCAATTCTCGATTTGTGCG
Universal reverse		GTGCAGGGTCCGAGGT

## Data Availability

Small RNA-seq data are deposited in GEO under accession GSE307378.

## Supplementary Materials

The supporting information can be downloaded at https://www.mdpi.com/article/10.3390/nu17182919/s1, File S1: ID miRNAs table. File S2: DEG miRNAs. File S3: psRNAtarget prediction.
